# Tuberculosis Pavilions at Isolation Hospitals

**Published:** 1914-09-26

**Authors:** 


### TUBERCULOSIS PAVILIONS AT ISOLATION HOSPITALS.

### Problems Involved in the Present Arrangements.

Reference was made in a recent issue of The Hospital to the refusal by a committee of the Taunton Council of sanction to the County Council scheme for the erection of pavilions for the treatment of tuberculous cases at the Taunton Isolation Hospital. A suggestion was made of further consideration and discussion. The fact is acknowledged that the treatment of tuberculous cases under similar arrangements is being undertaken elsewhere; also that a "saving of money" is to be anticipated—undoubtedly an attractive point.

With the pressing need which arose somewhat suddenly for accommodation for tuberculous patients, and with the transference to public bodies of the duty of finding such accommodation, all existing facilities were eagerly considered: the isolation and smallpox hospitals, which stood in the exceptional position of frequently having empty beds, were at an early stage seen to hold out possibilities. But little consideration was, however, needed to show that difficulties are many—difficulties of finance, of administration, and as to possibilities of infection.

The financial difficulties depend once again upon that bugbear of modern local government—divided authority. What (in such an arrangement) is to be provided by the hospital committee and what by the County Council, what is to be paid by the latter, what undertakings are to be given for the continuance of the arrangement? When a hospital committee has unoccupied buildings, necessary outfit, and separate staff complete, and is in a position to make a simple offer to receive, board, and nurse during a fixed term at a fixed rate tuberculous patients only, the matter is a comparatively simple one. Very different is the position in the case of the erection of buildings by an authority upon land belonging to another and adjoining a fever hospital, with the secondary questions of heating and lighting, furnishing, maintenance of patients, medical treatment, nursing, attendance, and many minor considerations. In this instance the bargain becomes at once a complicated one, full of liabilities from which either party to it may find it impossible to withdraw, however great the dissatisfaction which may arise or whatever changes of plans may later become necessary.

The financial complications merge into the administrative, just as those again are involved with the questions of treatment and of infection; there is no hard-and-fast border-line. Into all the difficulties of such a scheme are therefore drawn, each in degree, the medical officer of the hospital, the medical officer of health (especially if he be also the hospital medical officer), the tuberculosis medical officer, the hospital nursing staff, and the local medical practitioners. Of course friction should not arise between the medical men concerned; equally of course, there being ample opportunity for friction, it will arise in some instances. The position of neither the hospital medical officer nor the tuberculosis medical officer is, however, untenable. Whatever qualifications the former (whether resident or not) may possess, he is unlikely to have been appointed upon the strength of exceptional experience in the treatment of tuberculosis. He may none the less be asked to treat and supervise the patients, although not to carry out full sanatorium régime, and, if he be then ready and willing to accept the tuberculosis medical officer as a visiting and consulting officer and customarily to leave for his decision such matters as the duration of patients' stay, things may, and should, work smoothly. As in most official work momentous issues will arise, and tact and mutual consideration will be needed in what must finally often be a dual control. The staff must be under the control of the hospital medical officer, and such disciplinary difficulties as may arise in connection with the new work amongst the nursing and general hospital staff are unlikely to be more than temporary and could be overcome with the assistance of an efficient matron working under an intelligent committee.

It is clear that of the different classes of tuberculous patients for which accommodation has to be provided it is for the "hospital" class that such provision as is now being considered would be made. The more advanced cases of disease would be those received; and of them a large proportion would be patients in bed ("heavy" nursing cases) and patients with incessant cough and profuse sputa, all of whom need special and careful dieting and much care and supervision. Objections have been raised to the segregation and nursing of such patients in separate blocks of a fever hospital. Such objections cannot, however, be upheld as prohibitive or even serious; many of them apply to the commonly existing arrangements for nursing different specific fevers in isolation hospitals, and have been proved to be in practice negligible. In fact, they are disproved by the interesting recent experiments in bed-isolation or case-isolation in isolation hospital treatment. The conveyance of tuberculous infection from ward to ward under similar conditions, whilst its possibility cannot be disproved, is practically unlikely. Even making allowance for the fact that "hospital" cases of advanced type are the most likely to be infectious, especially to those nursing them or in immediate and constant contact with them, there is no evidence (statistical or other) that this infectivity is high. Such cases have been, and are, treated in hospitals and Poor-Law infirmaries, under conditions often far from ideal, without proof of actual danger even to the nursing staff. (The note upon the inquiries of Hamel, of Berlin, in The Hospital for June 20 bears upon this question and contains the very interesting remark that "in hospitals of old design the evidence of infection was as much as twice that in new, up-to-date institutions.") The Departmental Committee considered separate segregation unnecessary under proper conditions.

